# Sugar-sweetened beverages and the risk of hyperuricemia and gout: a meta-analysis

**DOI:** 10.3389/fnut.2025.1669129

**Published:** 2025-10-20

**Authors:** Yuejie Lu, Yinuo Wang, Renjie Huang, Hejing Pan, Zhijun Xie, Chengping Wen, Lin Huang, Xuanlin Li

**Affiliations:** ^1^The First Affiliated Hospital of Zhejiang Chinese Medical University (Zhejiang Provincial Hospital of Chinese Medicine), Hangzhou, China; ^2^The Second Clinical Medical School, Zhejiang Chinese Medical University, Hangzhou, China; ^3^School of Basic Medicine Sciences, Zhejiang Chinese Medical University, Hangzhou, China

**Keywords:** sugar-sweetened beverages, hyperuricemia, gout, fructose, meta-analysis, observational studies

## Abstract

**Objective:**

This meta-analysis aimed to investigate the association between sugar-sweetened beverages (SSBs), fructose, and the risk of gout and hyperuricemia.

**Methods:**

Following PRISMA guidelines, we systematically searched PubMed, EMBASE, and Cochrane Library for observational studies from inception to March 2025. Adjusted odds ratios (ORs) with 95% confidence intervals (CIs) were pooled using random/fixed-effects models. Subgroup analyses explored sex. Heterogeneity (*I*^2^) and publication bias were assessed.

**Results:**

A total of 22 studies (235,790 participants) were included. SSB intake significantly increased the risk of hyperuricemia (OR = 1.33, 95% CI: 1.23–1.44) and gout (OR = 1.21; 95% CI 1.11–1.32). Fruit juice (FJ) showed a modest association with hyperuricemia (OR = 1.15, 95% CI: 1.02–1.29) and an increased risk of gout (OR = 1.28; 95% CI 0.96–1.72). Fructose consumption was strongly associated with increased gout risk (OR = 1.66, 95% CI: 1.27–2.18), but its relationship with hyperuricemia was inconsistent (OR = 1.12, 95% CI: 0.85–1.46). DSD showed a modest association with gout (OR = 1.14; 95% CI 0.95–1.35). Subgroup analysis revealed SSB and FJ consumption associated with elevated risks of hyperuricemia in males (SSBs: 1.37; FJ: 1.15) compared to females (SSBs: 1.29; FJ: 1.13).

**Conclusions:**

SSB consumption is associated with increased risks of hyperuricemia and gout, particularly in males.

**Systematic review registration:**

https://www.crd.york.ac.uk/PROSPERO/display_record.php?RecordID=1040227, PROSPERO (Unique Identifier: CRD420251040227).

## 1 Introduction

Gout is a metabolic disorder characterized by hyperuricemia which is defined as a serum urate level exceeding 7.0 mg/dl in men and 5.7 mg/dl in women (1). This condition leads to the formation of monosodium urate crystals, resulting in painful recurrent flares and tissue damage (2–4). The prevalence of gout varies globally, ranging from < 1 to 6.8%, with an incidence of 0.58−2.89 cases per 1,000 person-years (5). In addition to its acute symptoms, gout and hyperuricemia are strongly associated with a range of comorbidities, including hypertension, diabetes mellitus, metabolic syndrome, and cardiovascular diseases (6). Whilst gout can be eradicated with urate-lowering therapy and reduce the risk of recurrence by controlled diet, treatment options remain suboptimal (7, 8). Various factors contribute to the development of gout, including comorbidities, genetic pre-disposition, obesity, environmental influences, and dietary habits (6, 7, 9). Among these, diet plays a critical role in the onset and progression of the disease. Excessive consumption of sugar, particularly fructose, places an additional strain on the kidneys and has been linked to the development of chronic kidney disease, which in turn increases the risk of gout and hyperuricemia (10).

Sugar-sweetened beverages (SSBs) are a major source of added sugars in the diet (11). From 1990 to 2018, the global consumption of SSBs among children and adolescents increased by 23% (12), and a model estimated that 184,000 deaths/year worldwide attributable to SSB consumption (13). There is compelling evidence that the consumption of SSBs is directly associated with the risk of cardiovascular disease, cancer, neurological disorders, ectopic fat accumulation, and endocrine/metabolic outcomes (14, 15).

SSB consumption is increasingly recognized to increase the risk of gout and hyperuricemia. A meta-analysis of prospective studies noted an adverse association between SSB and juice intake and incident gout. However, the scope of the analysis was limited to a smaller number of studies (16). Another meta-analysis established a significant positive association between SSB intake and the risk of gout and hyperuricemia, though its inclusion of literature remains constrained (17). To address these limitations, we therefore performed the updated meta-analysis additional study with a new collection of 11 newly published studies after 2017, aimed to overcomes the challenges of small study inclusion, thereby enhancing the reliability and comprehensiveness of the findings, and to fill the gap in quantitative evidence regarding the impact of SSBs and dietary fructose as modifiable risk factors for hyperuricemia and gout.

## 2 Method

The meta-analysis followed the guidelines of the Preferred Reporting Items for Systematic Reviews and Meta-Analyses (PRISMA) (18). The protocol was pre-registered with the International Prospective Register of Systematic Reviews (PROSPERO) under the approval number CRD420251040227.

### 2.1 Data sources

A comprehensive search of PubMed, EMBASE, and the Cochrane Library databases was conducted up to March 7, 2025. The search was performed by two independent investigators using both Medical Subject Headings (MeSH) and keywords. The terms included “Sugar-Sweetened Beverages,” “Artificially Sweetened Beverages,” “Fructose,” “Gout,” and related outcomes such as “Hyperuricemia” and “Uric Acid.” Search details are provided in Supplementary Tables S1–S3.

### 2.2 Eligibility criteria

We included observational studies that were (1) case-control, cohort, or cross-sectional in design, and (2) investigated the relationship between sugar-sweetened beverages (SSBs) or fructose and the risk of gout or hyperuricemia.

Studies were excluded if they did not report relative risks (RRs), hazard ratios (HRs), or odds ratios (ORs) with corresponding 95% confidence intervals (CIs), as well as reviews, conference abstracts, case reports, editor letters, duplicate publications, and studies without relevant outcomes.

### 2.3 Study selection

Two reviewers (YN Wang and YJ Lu) independently screened the titles and abstracts of articles, excluding duplicates and irrelevant studies. Full texts of potentially eligible studies were retrieved and assessed for eligibility. Disagreements were resolved by a third reviewer (XL Li).

### 2.4 Data extraction

Data extraction was performed independently by the two aforementioned reviewers (YN Wang and YJ Lu), following systematic review guidelines (19). Pre-designed forms were used to extract data on the first author, year of publication, country, age, sex, sample size, study type, exposure and outcome assessments, and follow-up duration. Any discrepancies were resolved through discussion with XL Li.

### 2.5 Risk of bias

The Agency for Healthcare Research and Quality (AHRQ) checklist (https://www.ncbi.nlm.nih.gov/books/NBK35156/) was used to assess the quality of cross-sectional studies, categorizing them as low (< 4 “yes” responses), medium (4–6 “yes” responses), or high (>7 “yes” responses).

For longitudinal studies, the Newcastle-Ottawa Scale (NOS; http://www.ohri.ca/programs/clinical_epidemiology/oxford.asp) was used, with studies classified as low (0–3), moderate (4–6), or high (7–9) quality based on their star ratings.

### 2.6 Statistical analysis

We extracted adjusted ORs, RRs, HRs, and 95% CIs to evaluate the associations between exposures and outcomes. Given design heterogeneity, we treated HR/RR as relative risk proxies and OR as a prevalence ratio under low-prevalence assumptions (20, 21). Heterogeneity was assessed using Cochran' χ^2^ test (*P* < 0.1) and *I*^2^ statistic (*I*^2^ > 50% indicating substantial heterogeneity). For low heterogeneity (*P* ≥ 0.1, *I*^2^ ≤ 50%), fixed-effects models (Mantel–Haenszel) were used, while random-effects models (DerSimonian–Laird) were applied for moderate/high heterogeneity. Sensitivity analyses excluded studies based on design to test robustness, and subgroup analyses were performed by sex and design to explore heterogeneity. Publication bias was evaluated using funnel plot inspection and Egger's regression (*P* < 0.05). Statistical analyses were conducted using Stata 14.0 (Stata Corp, TX, USA).

## 3 Result

### 3.1 Study selection

**Figure 1:**
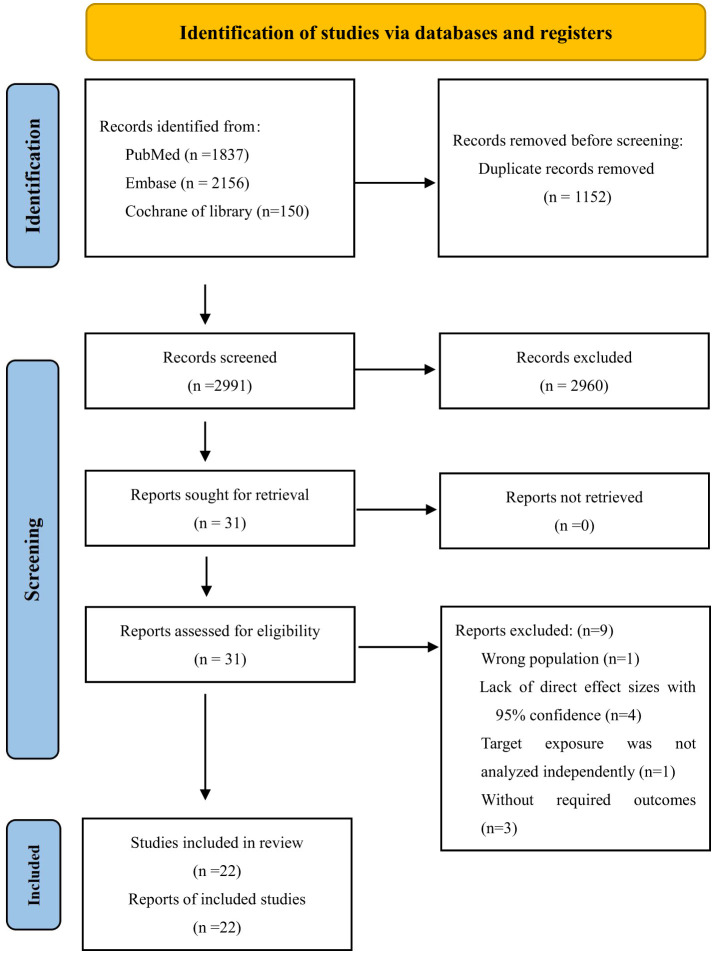
PRISMA flowchart of study selection.

Our search strategy identified 4,143 articles. After removing 1,152 duplicates, 2,991 abstracts were reviewed in detail. Of these, 2,960 were excluded based on title and abstract screening. Among the remaining 31 studies, nine were further excluded for the following reasons: one study reported an incorrect population, four lacked effect values or CIs, one did not analyze the target exposure independently, and three did not meet the eligibility criteria. Ultimately, 22 studies were included in the systematic review (22–43). A flowchart of the selection process is shown in Figure 1. Excluded studies and their reasons are listed in Supplementary Table S4.

### 3.2 Study characteristics

This meta-analysis included 22 studies, with a total of 235,790 participants, published up to January 12, 2025. The studies were diverse: 14 cross-sectional, seven prospective cohort, and one case-control. Most assessed SSB and fructose intake using validated FFQs, while some relied on 24-h recalls or self-reported questionnaires. Diagnostic criteria for hyperuricemia and gout were well-defined across most studies. Follow-up durations ranged from 2 to 22 years. Key confounders adjusted for included age, sex, BMI, energy intake, alcohol, hypertension, diabetes, and dietary factors. The studies represented a global population, with participants from Asia (China, South Korea, and Singapore), North America (USA, Mexico), South America (Brazil), and Oceania (New Zealand). Key study characteristics are summarized in Table 1.

**Table 1:** Characteristics of the included studies.

**Author**	**Year**	**Country**	**Age (Mean ±SD)**	**Sex**	**Sample size**	**Study type**	**Exposure**	**Exposure assessment**	**Outcome**	**Outcome assessment**	**Follow-up years**
**SSB and the risk of gout and hyperuricemia (cohort study)**											
Choi et al.	2008	USA	40–75	M	46,393	Cohort study	SSB DSD FJ	FFQ	Gout	ACR criteria	12
Choi et al.	2010	USA	30–55	F	78,906	Cohort study	SSB DSD FJ	FFQ	Gout	ACR criteria	22
Bomback et al.	2010	USA	45–64	M/F	9,451	Cohort study	SSB	FFQ	Hyperuricemia	NR	3
Meneses-León et al.	2020	Mexico	18–85 (45.8 ± 12.9)	M/F	1,300 F: 978 M: 322	Cohort study	SSB, DSD	FFQ	Hyperuricemia	The uricase colorimetric method	14
Siqueira et al.	2021	Brazil Mexico	35–74 (51.3 ± 8.9)	M/F	10,072 M: 4,151 F: 5,921	Cohort study	SSB FJ	FFQ	Hyperuricemia	The uricase colorimetric method	4
Rai et al.	2024	USA	M: 53.8 ± 9.8 F: 50.9 ± 7.2	M/F	122,679 M: 43,703 F: 78,976	Cohort study	SSB FJ	FFQ	Gout	ACR criteria	26
Zhang et al.	2025	China	22–87 (48.48 ± 11.82)	M/F	10,883 M: 6,449 F: 4,434	Cohort study	SSB	Simple Question	Hyperuricemia	Laboratory	2
**SSB and the risk of gout and hyperuricemia (case-control study)**											
Batt et al.	2014	New Zealand European Caucasian	57.9 (17–94) Case: 63.8 (23–94) Control: 44.4 (17–79)	M/F	592 Case: 412 Control: 18	Case-control study	SSB	Simple Question	Gout	Self reported Physician diagnosed	/
		New Zealand (Maori)	47.1 (17–81) Case: 54.2 (23–81) Control: 42.7 (17–80)		502 Case: 190 Control: 312						
		New Zealand (Pacific Islander)	44.6 (17–86) Case: 47.7 (18–81) Control: 39.9 (17–6)		540 Case: 323 Control: 217						
		Atherosclerosis Risk In Communities	53.8 (44–5) Case: 4.5 (45–5) Control: 3.8(44–5)		7,075 Case: 148 Control: 6,927						
**SSB and the risk of gout and hyperuricemia (cross-sectional study)**											
Dalbeth et al.	2015	New Zealand	49.5	M/F	2,578	Cross-sectional study	SSB	Self-reported	Gout	Clinically ascertained by ARA	/
Bomback et al.	2010	USA	45–64	M/F	15,745	Cross-sectional study	SSB	FFQ	Hyperuricemia	NR	/
Teng et al.	2013	Singapore	45–74 (57.6 ± 7.9)	M/F	483	Cross-sectional study	SSB	FFQ	Hyperuricemia	Direct enzymatic assay	/
Bae et al.	2014	Korea	M: 62.5 ± 9.6 F: 61.6 ± 9.8	M/F	9,400 M: 3,564 F: 5,836	Cross-sectional study	SSB	FFQ	Hyperuricemia	Laboratory	/
Meneses-Leon et al.	2014	Mexico	18–70 M: 42.6 ± 11.6 F: 43.0 ± 12.2	M/F	6,705 M: 1,956 F: 4,749	Cross-sectional study	SSB	FFQ	Hyperuricemia	Enzymatic Colorimetric method	/
Li et al.	2022	China	42.7 ± 14.2	M	620	Cross-sectional study	SSB	FFQ	Hyperuricemia	Laboratory	/
Lin et al.	2013	China	12–16	M/F	2,727	Cross-sectional study	SSB	FFQ	Hyperuricemia	Modified hexokinase enzymatic method	/
Lee et al.	2024	Korean	19–64	M/F	2,881 M: 1,066, F: 1,815	Cross-sectional Study	SSB	FFQ	Hyperuricemia	The uricase colorimetric method	/
Siqueira et al.	2018	Brazil Mexico	35–74 50 ± 8.4	M/F	7,173 M: 3,325, F: 3,848	Cross-sectional study	SSB FJ	FFQ	Hyperuricemia	The uricase colorimetric method	/
Lee et al.	2021	Korea	53.1 ± 8.3	M/F	167,752	Cross-sectional study	SSB	Simple question	Hyperuricemia	NR	/
Zhang et al.	2020	China	>18	M	25,507 M: 13,013 F: 12,494	Cross-sectional study	SSB	FFQ	Hyperuricemia	Enzymatic colorimetric test	/
Lin et al.	2021	USA	≥18	M	15,338 M: 7,580 F: 7,758	Cross-sectional study	SSB	24-h dietary recall	Hyperuricemia	NR	/
So et al.	2020	Korea	47 ± 16.4	M	10,175 M: 4,300 F: 5,875	Cross-sectional Study	SSB	24-h dietary recall	Hyperuricemia	NR	/
**Fructose and the risk of gout and hyperuricemia (cohort study)**											
Choi et al.	2008	USA	40–75	M	755	Cohort study	Fructose	FFQ	Gout	ACR criteria	12
Choi et al.	2010	USA	30–55	F	78,906	Cohort study	Fructose	FFQ	Gout	ACR criteria	22
**Fructose and the risk of gout and hyperuricemia (cross-sectional study)**											
Sun et al.	2010	USA	20–80	M/F	9,384	Cross-sectional study	Fructose	24-h dietary recall	Hyperuricemia	The uricase colorimetric method	/
Siqueira et al.	2018	Brazil Mexico	35–74	M/F	7,173 M: 3,325 F: 3,848	Cross-sectional study	Fructose	FFQ	Hyperuricemia	The uricase colorimetric method	/
Zheng et al.	2021	USA	21–91	M/F	4,576	Cross-sectional study	Fructose	FFQ	Hyperuricemia	The uricase colorimetric method	/

M, Male; F, Female; SSB, Sugar-sweetened beverage, included sugar sweetened sodas, sugar-containing carbonated beverages, and sugar-sweetened products; DSD, diet soft drinks; FJ, fruit juices; FFQ, Food Frequency Questionnaire; ACR, American College of Rheumatology; ARA, American Rheumatology Association.

### 3.3 Quality assessment

Cross-sectional studies had an average AHRQ score of 7.81/11 (Supplementary Table S5), and longitudinal studies scored 7.63/9 on the NOS, indicating overall good quality (Supplementary Table S6).

### 3.4 SSB and hyperuricemia risk

**Figure 2:**
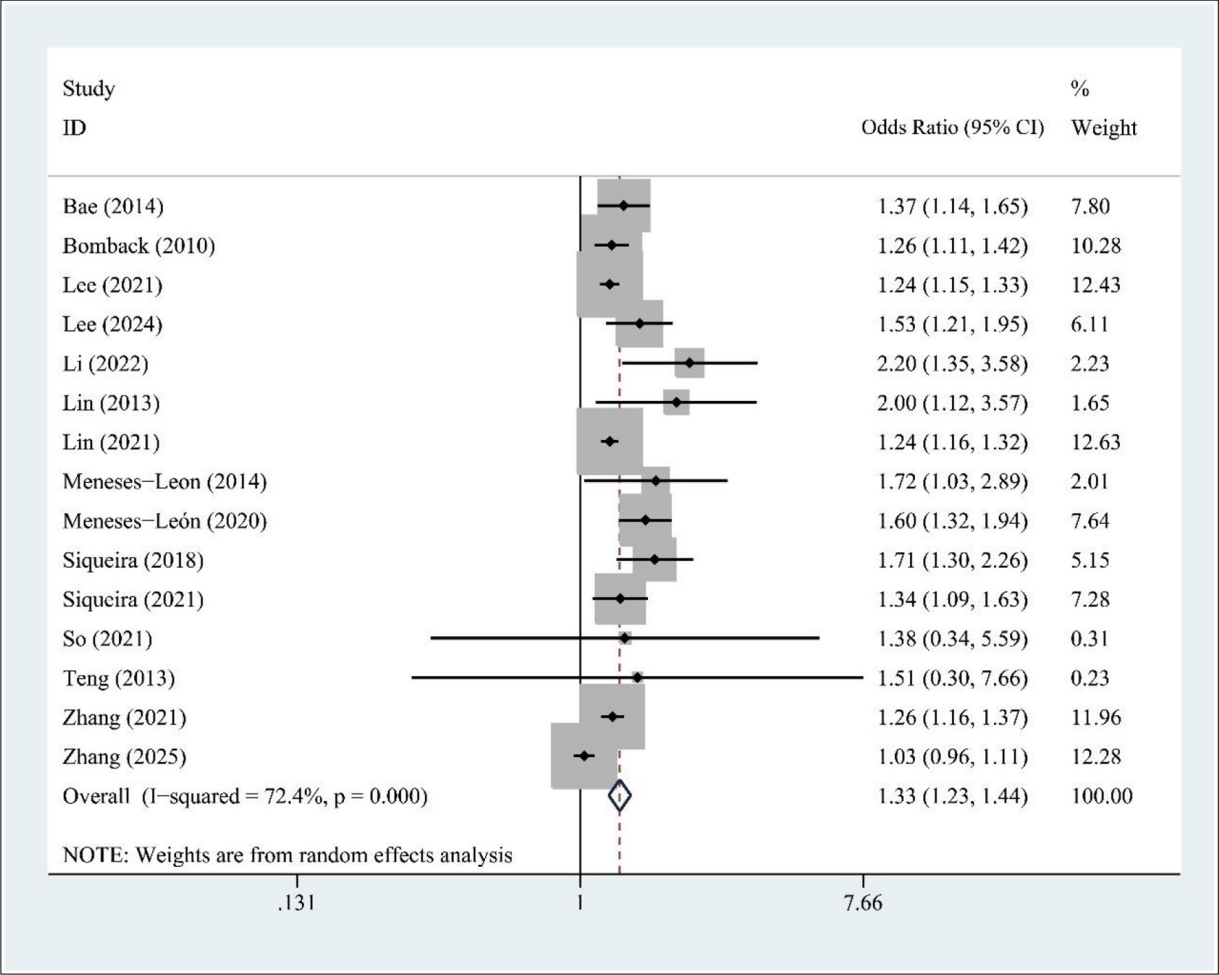
Forest plot of the association between sugar-sweetened beverage (SSB) consumption and hyperuricemia risk.

The meta-analysis of 15 studies found a significant association between SSB consumption and increased hyperuricemia risk [OR = 1.33, 95% CI (1.23, 1.44), *I*^2^ = 72.4%, *P* = 0.000] (Figure 2). Despite high heterogeneity, sensitivity analysis confirms result stability (Supplementary Figure S1).

### 3.5 FJ and hyperuricemia risk

**Figure 3:**
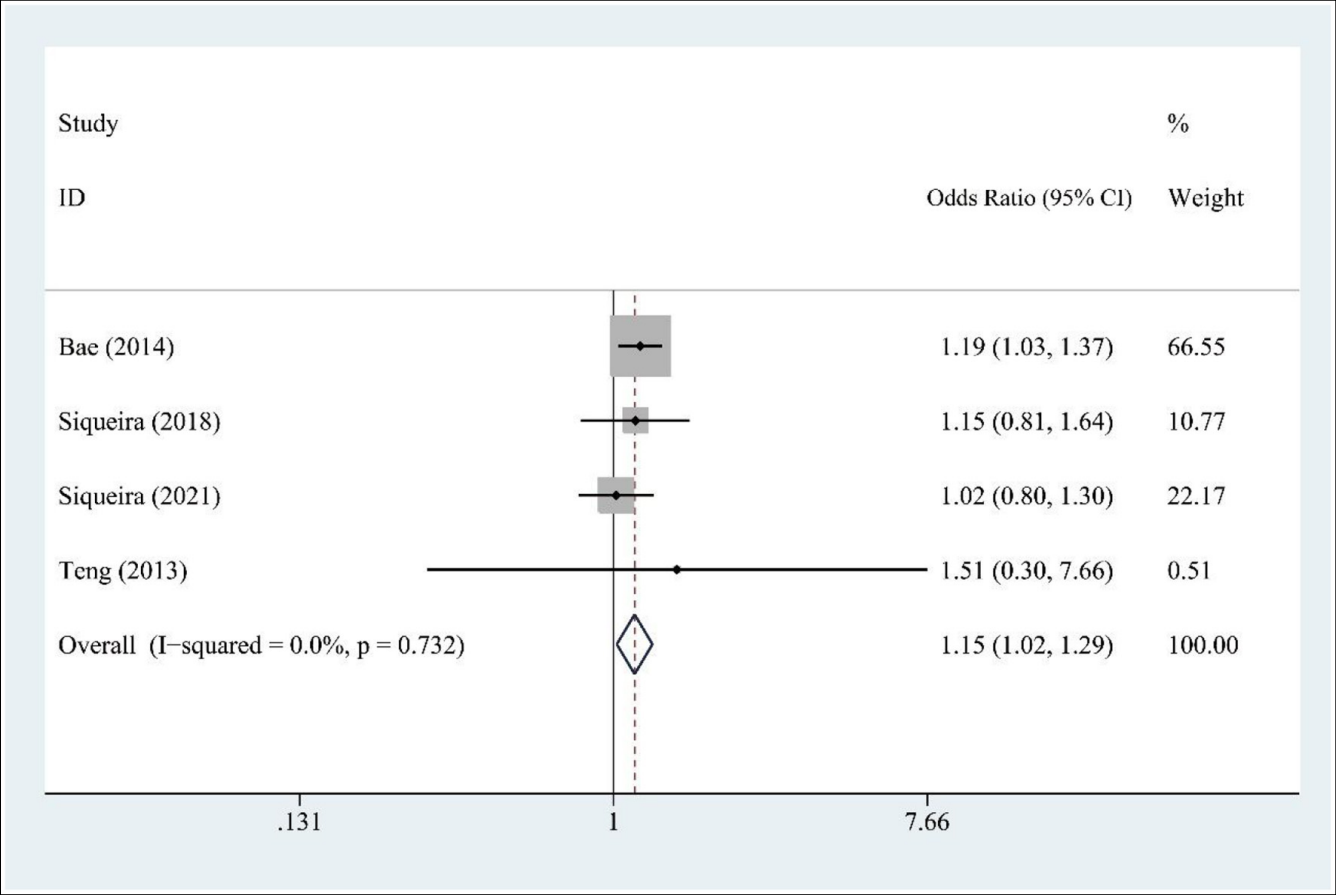
Forest plot of the association between fruit juice (FJ) consumption and hyperuricemia risk.

Fruit juice (FJ) intake (four studies) was modestly associated with hyperuricemia risk [OR = 1.15, 95% CI (1.02, 1.29), *I*^2^ = 0.0%, *P* = 0.732] (Figure 3).

### 3.6 Fructose and hyperuricemia risk

**Figure 4:**
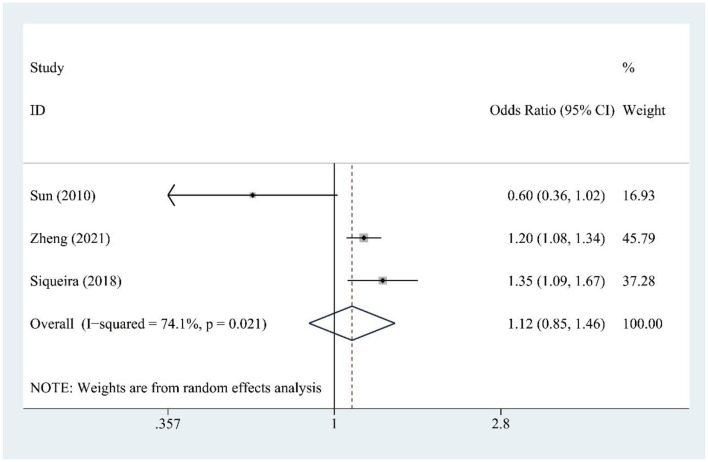
Forest plot of the association between fructose consumption and hyperuricemia risk.

Fructose consumption (three studies) was not significantly associated with hyperuricemia risk [OR = 1.12, 95% CI (0.93, 1.85), *I*^2^ = 74.1%, *P* = 0.021] (Figure 4). Moderate heterogeneity indicates some variability. Sensitivity testing showed no substantial changes in the findings (Supplementary Figure S2).

### 3.7 SSB and gout risk

**Figure 5:**
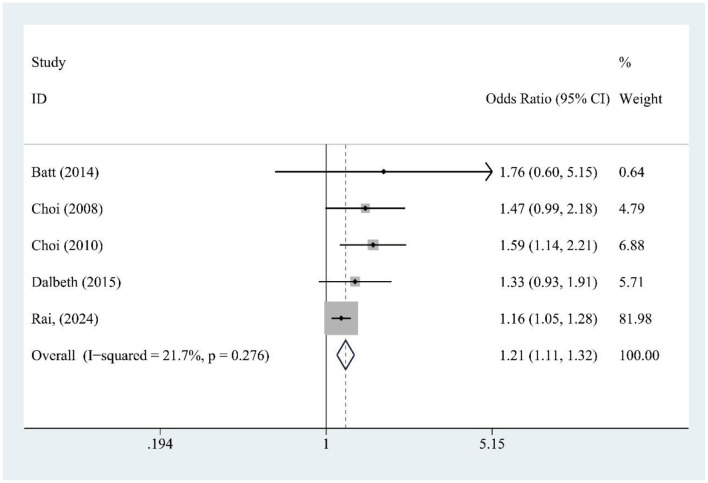
Forest plot of the association between sugar-sweetened beverage (SSB) consumption and gout risk.

SSB consumption (five studies) was significantly associated with increased gout risk [OR = 1.21, 95% CI (1.11, 1.32), *I*^2^ = 21.7%, *P* = 0.276] (Figure 5), with low heterogeneity supporting consistent findings.

### 3.8 FJ and gout risk

**Figure 6:**
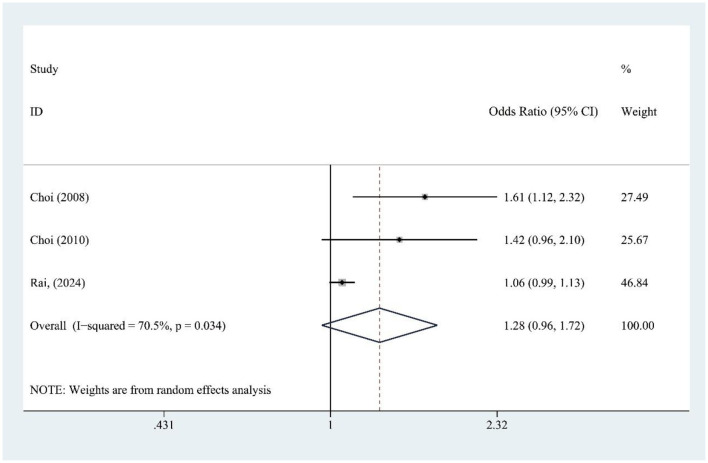
Forest plot of the association between fruit juice (FJ) consumption and gout risk.

FJ intake (three studies) was not significantly associated with gout risk [OR = 1.28, 95% CI (0.96, 1.72), *I*^2^ = 70.5%, *P* = 0.034] (Figure 6), though high heterogeneity suggests variability among studies. Sensitivity analysis confirms result stability (Supplementary Figure S3).

### 3.9 DSD and gout risk

**Figure 7:**
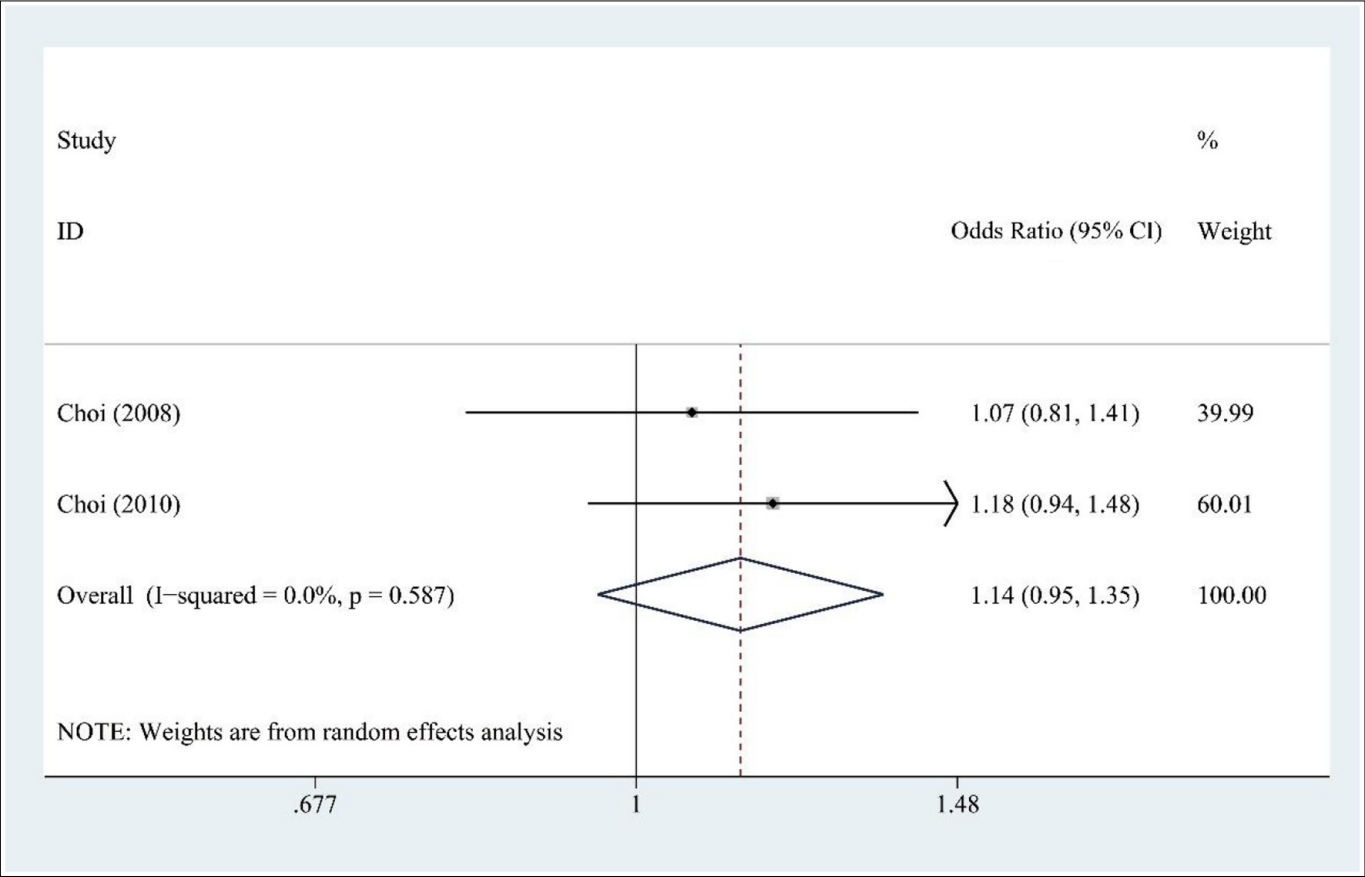
Forest plot of the association between diet soft drink (DSD) consumption and gout risk.

DSD consumption (two studies) showed no significant association with gout risk [OR = 1.14, 95% CI (0.95, 1.35), *I*^2^ = 0.0%, *P* = 0.587] (Figure 7), with no heterogeneity indicating reliable findings.

### 3.10 Fructose and gout risk

**Figure 8:**
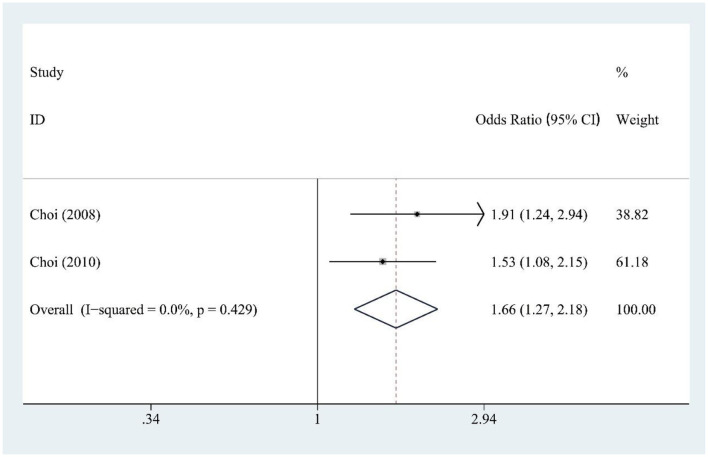
Forest plot of the association between fructose consumption and gout risk.

Fructose intake (two studies) was significantly associated with increased gout risk [OR = 1.66, 95% CI (1.27, 2.18), *I*^2^ = 0.0%, *P* = 0.429] (Figure 8), showing a strong and consistent effect across studies.

### 3.11 Subgroup analysis

Among females (eight studies), SSB intake was significantly associated with higher hyperuricemia risk [OR = 1.29, 95% CI (1.10, 1.50), *I*^2^ = 37.2%, *P* = 0.132] (Table 2). Males (eight studies) showed a stronger association [OR = 1.37, 95% CI (1.16, 1.63), *I*^2^ = 80.1%, *P* = 0.000] (Table 2).

**Table 2:** Subgroup analysis for the risk of SSB and hyperuricemia and gout.

**Subgroups**	**Included studies**	**OR (95% CI)**	**Heterogeneity**	
			*I*^2^ **(%)**	***P*** **-values**
**Sex (SSB and hyperuricemia)**				
Female	8	1.29 (1.10–1.50)	37.2	0.132
Male	8	1.37 (1.16–1.63)	80.1	0.000
**Sex (FJ and hyperuricemia)**				
Female	3	1.13 (0.95–1.35)	13.4	0.315
Male	3	1.15 (0.97–1.38)	0.0	0.721

SSB, Sugar-sweetened beverage; FJ, Fruit juices.

For FJ, no significant association with hyperuricemia risk was found in either females (three studies) [OR = 1.13, 95% CI (0.95, 1.35), *I*^2^ = 13.4%, *P* = 0.315] or males (three studies) [OR = 1.15, 95% CI (0.97, 1.38), *I*^2^ = 0.0%, *P* = 0.721] (Table 2).

### 3.12 Publication bias

For SSB and hyperuricemia, Begg's test showed no significant bias (*P* = 0.181), but Egger's test suggested small-study effects (*P* = 0.012; Supplementary Figure S4A). No bias was detected for FJ (*P* = 1.000, *P* = 0.243) or fructose (*P* = 1.000, *P* = 0.279; Supplementary Figures S4B, C). Publication bias for SSB and gout was inconclusive, with marginal significance for bias in Egger's test (*P* = 0.047; Supplementary Figure S5A). No significant bias was found for FJ and gout (Supplementary Figure S5B), and DSD and gout (Supplementary Figure S5C). Fructose and gout analysis was inconclusive due to the small sample size (Supplementary Figure S5D).

## 4 Discussion

### 4.1 Main findings

The findings of this meta-analysis of 235,790 participants suggest that SSB consumption is at significantly increased risks of hyperuricemia and gout, and the risk is higher in males than in females. The findings also emphasize the need for early intervention and continuous monitoring of people with large SSB consumption.

### 4.2 Comparison with previous meta-analyses

In recent years, increasing numbers of observational studies have reported the increased risk of hyperuricemia and gout in people with SSB consumption. Previous meta-analysis (16), based on three studies, concluded that a history of SSB consumption was associated with a 1.77 (1.20–2.61; *I*^2^ = 0.0%) times increased risk of incident gout, and FJ consumption was associated with a 2.08 (1.40–3.08; *I*^2^ = 0.0%) times increased risk of incident gout. However, the number of studies included in this study was too small and did not explore the association between SSBs and hyperuricemia. Another meta-analysis (17) based on nine studies, showed SSB consumption was associated with a 1.35 (1.18–1.55, *I*^2^ = 40.1%) times increased risk of incident gout, a 1.33 (1.06–1.66, *I*^2^ = 0.0%) times increased risk of prevalent gout, and a 1.35 (1.19–1.52, *I*^2^ = 41.4%) times increased risk of hyperuricemia. But this study did not analyze SSBs separately from FJ. In this meta-analysis, we found that consumption of SSBs was associated with a 1.33-fold increased risk of hyperuricemia and a 1.21-fold increased risk of gout. Consumption of FJ was linked to a 1.15-fold increased risk of hyperuricemia and a 1.28-fold increased risk of gout. Additionally, we analyzed that fructose consumption was associated with a 1.12-fold increased risk of hyperuricemia and a 1.66-fold increased risk of gout. Also, DSD consumption was found to have a 1.14-fold increased risk of gout. The subgroup analysis indicated that the risk of SSBs and FJ for hyperuricemia was higher in male.

This study had several advantages over previous meta-analysis. Firstly, our study incorporated the most recent and most comprehensive data to obtain a more complete understanding of the associations. Secondly, we examined the risk of developing hyperuricemia and gout for SSBs, FJ, fructose, and DSD consumption separately, with subgroup analyses by gender.

### 4.3 Potential underlying mechanisms

SSB-associated hyperuricemia and gout risks arise through interconnected metabolic pathways. Glucose from SSBs fuels purine synthesis via the pentose phosphate pathway, increasing phosphoribosyl pyrophosphate availability for nucleotide formation (44). Excessive glucose intake—a cardinal manifestation of nutrient surplus—activates mTOR/HIF-1α signaling. This dual activation drives anabolic shifts and oxidative stress, while simultaneously inducing insulin resistance. Collectively, these perturbations culminate in proximal tubule dysfunction, thereby impairing renal urate excretion (45–47).

Fructose uniquely drives hyperuricemia through direct hepatic metabolism. Its phosphorylation by fructokinase generates fructose-1-phosphate (F1P), depleting adenosine triphosphate (ATP) and inorganic phosphate (Pi). This impairs adenosine diphosphate (ADP) phosphorylation, trapping F1P while adenylate kinase converts accumulating ADP to adenosine monophosphate (AMP)—a direct uric acid precursor. Concurrently, ATP/Pi depletion attenuates feedback inhibition of urate production, amplifying hyperuricemia (33, 48). While chronic intake downregulates urate excretion transporters thereby reducing uric acid excretion (49). Emerging evidence implicates fructose-mediated gut microbiota alterations in gout pathogenesis (50), suggesting novel therapeutic targets. These mechanisms underscore the importance of SSB reduction in metabolic disorder prevention strategies.

Women have a higher rate of urate excretion and a lower rate of post-secretory reabsorption of renal tubular urate compared to men (51), which may be due to the effect of estrogen (52). This evidence is consistent with the results of our subgroup analysis.

### 4.4 Clinical recommendations

SSB intake is associated with a higher risk of hyperuricemia and gout, especially in males. Clinically, it is common to routinely inquire about the intake history of SSBs in patients with obesity, hypertension, and chronic kidney disease. In combination with the WHO and WCRF/AICR recommendations and our findings, we recommend reducing the consumption of free sugars or added sugars to below 25 g/day and limiting the consumption of sugar sweetened beverages to less than one serving a week (approximately 200–355 ml/week). Nevertheless, adherence to this precise threshold may be impractical across diverse socioeconomic and cultural contexts. A more feasible public health objective is to prioritize reduction in SSB consumption overall, as any decrease confers benefit. Achieving this requires complementary policy interventions—such as SSB taxation, marketing restrictions, improved nutrition labeling, and enhanced access to healthier beverages—to enable sustained population-level behavior change. To change sugar consumption patterns, especially for children and adolescents, a combination of widespread public health education and policies worldwide is urgently needed (14). In particular, male patients should be more strictly restricted from consuming SSBs due to a higher risk associated with gender differences.

### 4.5 Strengths and limitations

This meta-analysis robustly links SSB intake to hyperuricemia and gout risks across diverse populations (*n* = 235,790), with methodological strengths including PRISMA adherence, sensitivity analyses, and high-quality study inclusion. Limitations encompass: (1) causal inference constraints from 14 cross-sectional studies (of 22) due to potential reverse causality (e.g., post-diagnosis SSB reduction); (2) elevated heterogeneity likely attributable to variations in study design, exposure/outcome assessment, intake units, recall bias, diet/lifestyle factors, residual confounders, dietary patterns, and diagnostic criteria, potentially affecting results despite robust sensitivity analyses; (3) limited statistical power for DSD/fructose analyses, along with feasibility challenges in defining heterogeneous beverage categories, such as distinguishing between natural and added-sugar juices or artificial and non-caloric sweeteners, due to the necessitated reliance on prior classifications that may confound outcomes; and (4) geographic bias toward predominantly Asia and North America, non-uniform SSB assessment methods, and unspecified serum urate assays in some studies, all of which potentially contribute to publication bias. Evidence quality is anticipated to improve with future updates, more high-quality studies, broader geographic data, and standardized measurements.

## 5 Conclusions

SSB intake is associated with a higher risk of hyperuricemia, especially in males, and a modest increase in the risk of gout. FJ intake posed a slight risk for hyperuricemia and gout. Fructose intake was not significantly associated with hyperuricemia risk but significantly associated with gout risk. DSD intake posed a slight risk for gout. The results highlight the need to reduce and continuous monitor SSB intake, especially in high-risk populations.

## Data Availability

The original contributions presented in the study are included in the article/Supplementary material, further inquiries can be directed to the corresponding authors.
