# Supplementary material for: Sugar-sweetened beverages and the risk of hyperuricemia and gout: a meta-analysis
Source: Front Nutr. 2025 Oct 20;12:1669129. doi: 10.3389/fnut.2025.1669129 (PMC12580599; doi:10.3389/fnut.2025.1669129)
Supplement: Supplementary file 1 [file Table_1.docx]

**Supplementary Table 1-3: Details of the Literature Search Strategy**

Supplementary Table 1: PubMed

| No. | Content | Result |
| --- | --- | --- |
| #1 | Search:"fructose"[Mesh]Sort by: Most Recent | 19405 |
| #2 | Search: (Levulose[Title/Abstract]) OR (fructose[Title/Abstract])Sort by: Most Recent | 39280 |
| #3 | Search: "Sugar-Sweetened Beverages"[Mesh] Sort by: Most Recent | 1246 |
| #4 | Search: "Beverages"[Mesh] Sort by: Most Recent | 184789 |
| #5 | Search: "Fruit and Vegetable Juices"[Mesh]Sort by: Most Recent | 3385 |
| #6 | Search: "Carbonated Beverages"[Mesh] Sort by: Most Recent | 3709 |
| #7 | Search: "Artificially Sweetened Beverages"[Mesh] Sort by: Most Recent | 94 |
| #8 | Search: ("Beverage*, Sweet*"[Title/Abstract] OR "Beverage*,Sugar Sweet*"[Title/Abstract] OR "Sugar* Sweet* Beverage*"[Title/Abstract] OR "Sugar Sweet*"[Title/Abstract] OR "Sugar Added Beverage"[Title/Abstract] OR "Sugar Added Beverages"[Title/Abstract] OR "Sugar Add*"[Title/Abstract] OR "Sweet* Beverage*"[Title/Abstract] OR "Sweet* Drink*"[Title/Abstract] OR "Drink*, Sweet*"[Title/Abstract] OR "SSB"[Title/Abstract] OR "SSBs"[Title/Abstract] OR "ASB"[Title/Abstract] OR "Artificially sweetened soda"[Title/Abstract] OR "soft drink*"[Title/Abstract] OR "soft-drink*"[Title/Abstract] OR "fruit drink*"[Title/Abstract] OR "sport* drink*"[Title/Abstract] OR "energy drink*"[Title/Abstract] OR "caloric drink*"[Title/Abstract] OR "sugar* drink*"[Title/Abstract] OR "sugar* beverage*"[Title/Abstract] OR "Calori* beverage*"[Title/Abstract] OR "Calori* drink*"[Title/Abstract] OR "Fructose beverage*"[Title/Abstract] OR "soda*"[Title/Abstract] OR "soda-pop"[Title/Abstract] OR "Pop"[Title/Abstract] OR "Cola*"[Title/Abstract] OR "Lemonade*"[Title/Abstract] OR "Ice tea*"[Title/Abstract] OR "Fruit juice*"[Title/Abstract] OR "Fruit punch"[Title/Abstract] OR "Punch"[Title/Abstract] OR "cordials"[Title/Abstract] OR "Juice*"[Title/Abstract] OR "Fruitade*"[Title/Abstract] OR "Vitamin water*"[Title/Abstract] OR "Non-nutritive sweetened beverage*"[Title/Abstract] OR "NSB"[Title/Abstract])Sort by: Most Recent | 98535 |
| #9 | Search: #1 OR #2 OR #3 OR #4 OR #5 OR #6 OR #7 OR #8 | 310892 |
| #10 | Search: "Gout"[Mesh]Sort by: Most Recent | 14894 |
| #11 | Search: "Hyperuricemia"[Mesh] Sort by: Most Recent | 5980 |
| #12 | Search:"Uric Acid"[Mesh] Sort by: Most Recent | 30224 |
| #13 | Search: ("gouts"[Title/Abstract] OR "Hyperuricemi*"[Title/Abstract] OR "hyperuricaemi* "[Title/Abstract] OR "hyperuricacid*"[Title/Abstract] OR "Uric"[Title/Abstract] OR "uric acid*"[Title/Abstract] OR "urate"[Title/Abstract] OR "gout"[Title/Abstract]) Sort by: Most Recent | 61285 |
| #14 | Search: #10 OR #11 OR #12 OR #13 | 68515 |
| #15 | Search: #9 AND #14 | 1837 |

Supplementary Table 2 Embase

| No. | Content | Result |
| --- | --- | --- |
| #1 | 'sugar-sweetened beverage'/exp | 5153 |
| #2 | 'fructose'/exp | 32770 |
| #3 | 'gout'/exp | 32282 |
| #4 | 'hyperuricemia'/exp | 50667 |
| #5 | 'uric acid'/exp | 68857 |
| #6 | 'beverage*, sweet*':ab,ti OR 'beverage*,sugar sweet*':ab,ti OR 'sugar* sweet* beverage*':ab,ti OR 'sugar sweet*':ab,ti OR 'sugar added beverage':ab,ti OR 'sugar added beverages':ab,ti OR 'sugar add*':ab,ti OR 'sweet* beverage*':ab,ti OR 'sweet* drink*':ab,ti OR 'drink*, sweet*':ab,ti OR 'ssb':ab,ti OR 'ssbs':ab,ti OR 'asb':ab,ti OR 'artificially sweetened soda':ab,ti OR 'soft drink*':ab,ti OR 'soft-drink*':ab,ti OR 'fruit drink*':ab,ti OR 'sport* drink*':ab,ti OR 'energy drink*':ab,ti OR 'caloric drink*':ab,ti OR 'sugar* drink*':ab,ti OR 'sugar* beverage*':ab,ti OR 'calori* beverage*':ab,ti OR 'calori* drink*':ab,ti OR 'fructose beverage*':ab,ti OR 'soda*':ab,ti OR 'soda-pop':ab,ti OR 'pop':ab,ti OR 'cola*':ab,ti OR 'lemonade*':ab,ti OR 'ice* tea*':ab,ti OR 'fruit juice*':ab,ti OR 'fruit punch':ab,ti OR 'punch':ab,ti OR 'cordials':ab,ti OR 'juice*':ab,ti OR 'fruitade*':ab,ti OR 'vitamin water*':ab,ti OR 'non-nutritive sweetened beverage*':ab,ti OR 'nsb*':ab,ti OR 'fructose':ab,ti OR 'levulose':ab,ti | 174454 |
| #7 | 'gouts':ab,ti OR 'hyperuricemi*':ab,ti OR 'hyperuricaemi*':ab,ti OR 'hyperuricacid*':ab,ti OR 'uric':ab,ti OR 'uric acid*':ab,ti OR 'urate':ab,ti OR 'gout':ab,ti | 86657 |
| #8 | #1 OR #2 OR #6 | 185157 |
| #9 | #3 OR #4 OR #5 OR #7 | 124198 |
| #10 | #8 AND #9 | 2156 |

Supplementary Table 3 Cochran Library

| No. | Content | Result |
| --- | --- | --- |
| #1 | MeSH descriptor: [Beverages] explode all trees | 8927 |
| #2 | MeSH descriptor: [Sugar-Sweetened Beverages] explode all trees | 126 |
| #3 | MeSH descriptor: [Carbonated Beverages] explode all trees | 226 |
| #4 | MeSH descriptor: [Artificially Sweetened Beverages] explode all trees | 10 |
| #5 | MeSH descriptor: [Fruit and Vegetable Juices] explode all trees | 460 |
| #6 | MeSH descriptor: [Fructose] explode all trees | 1209 |
| #7 | (“Sugar-Sweetened Beverages” OR “soft drink” OR “soft drinks” OR beverage OR beverages OR “carbonated soft drinks” OR fruitades OR “fruit drinks” OR “sports drinks” OR “vitamin water drink” OR “sweetened iced tea” OR punch OR “fruit punch” OR cordials OR lemonade OR soda OR soda-pop OR “Fruit Beverage” OR “Fruit drink” OR “fruit juice” OR “Beverages, Sugar-Sweetened” OR “Sugar-Sweetened Beverage” OR “Sugar Sweetened Beverage” OR “Beverages, Sugar Sweetened” OR “Beverage, Sugar Sweetened” OR “Sugar Sweetened Beverages” OR “Sweetened Beverages, Sugar” OR “Sweetened Beverage, Sugar” OR “Sugar-Added Beverage” OR “Beverages, Sugar-Added” OR “Beverage, Sugar-Added” OR “Sugar Added Beverage” OR “Sugar-Added Beverages” OR “Sugar Added Beverages” OR “Sweetened Beverages” OR “Sweetened Beverage” OR “Beverages, Sweetened” OR “Beverage, Sweetened” OR “Sweetened Drinks” OR “Sweetened Drink” OR “Drinks, Sweetened” OR “Drink, Sweetened” OR “Sugar-Sweetened Sodas” OR “Sugar Sweetened Sodas” OR “Sugar-Sweetened Soda” OR “Sodas, Sugar-Sweetened” OR “Soda, Sugar-Sweetened” OR “Sugar Sweetened Soda” OR “Sugar-Sweetened Soft Drinks” OR “Sugar Sweetened Soft Drinks” OR “Sugar-Sweetened Soft Drink” OR “Drinks, Sugar-Sweetened“ OR “Soft Drink, Sugar-Sweetened Soft” OR “Soft Drinks, Sugar-Sweetened” OR “Soft Drink, Sugar-Sweetened” OR “Sugar Sweetened Soft Drink” OR "SSB" OR "SSBs" OR "ASB" OR "Artificially sweetened soda" OR "fruit drinks" OR "sport drinks" OR "energy drink" OR "energy drinks" OR "caloric drinks" OR "sugar drinks" OR "sugar beverages" OR "Fructose beverages" OR "Pop" OR Cola* OR "Lemonades" OR "Ice tea" OR "Fruit juices" OR "Juice" OR "Juices" OR Fruitade OR “vitamin water” OR "Non-nutritive sweetened beverages" OR "NSB" OR "Calori beverages" OR "Calori drinks"):ab,ti,kw | 18396 |
| #8 | (“Fructose” OR “levulose”):ab,ti,kw | 1976 |
| #9 | #1 OR #2 OR #3 OR #4 OR #5 OR #6 #7 OR #8 | 25559 |
| #10 | (hyperuricemia):ab,ti,kw | 1578 |
| #11 | (gout):ab,ti,kw | 2158 |
| #12 | (gouts):ab,ti,kw | 5 |
| #13 | MeSH descriptor: [Hyperuricemia] explode all trees | 421 |
| #14 | MeSH descriptor: [Uric Acid] explode all trees | 421 |
| #15 | MeSH descriptor: [Gout] explode all trees | 620 |
| #16 | #10 OR #11 OR #12 OR #13 OR #14 OR #15 | 4112 |
| #17 | #9 AND #16 | 150 |

Table 4. Literature exclusion list

| No. | Title | Journal |
| --- | --- | --- |
| Wrong population (n=1) | | |
| 1 | Sugar Sweetened Beverage Consumption among Adults with Gout or Type 2 Diabetes | PLoS One |
| Lack of direct effect sizes with 95% CI (n=3) | | |
| 1 | Increased coffee, tea, or other sugar-sweetened beverage consumption in adolescents is associated with less satisfactory dietary quality, body fatness and serum uric acid profiles over the past 18 years in Taiwan | Asia Pacific Journal of Clinical Nutrition |
| 2 | High sugar-sweetened beverage intake frequency is associated with smoking, irregular meal intake and higher serum uric acid in Taiwanese adolescents | Journal of Nutritional Science |
| 3 | Pacific Islands Families Study: Serum Uric Acid in Pacific Youth and the Associations with Free-Sugar Intake and Appendicular Skeletal Muscle Mass | Nutrients |
| 4 | Sugar-sweetened soft drinks, diet soft drinks, and serum uric acid level: The Third National Health and Nutrition Examination Survey | Arthritis Care and Research |
| Target exposure was not analyzed independently (n=1) | | |
| 1 | Association of dietary patterns with blood uric acid concentration and hyperuricemia in northern Chinese adults | Nutrition Journal |
| Without required outcomes (n=3) | | |
| 1 | Sugar-sweetened beverages, serum uric acid, and blood pressure in adolescents | Journal of Pediatrics |
| 2 | Sweetened beverages intake, hyperuricemia and metabolic syndrome: the Mexico City Diabetes Study | Salud Publica de Mexico |
| 3 | Intake of Added Sugar and Sugar-Sweetened Drink and Serum Uric Acid Concentration in US Men and Women | Hypertension |

**Supplementary Table 5-6: Details of the AHRQ and NOS**

**Table 5.** The Agency for Healthcare Research and Quality (AHRQ*) checklist for cross‐sectional studies.

| **Study** | 1 | 2 | 3 | 4 | 5 | 6 | 7 | 8 | 9 | 10 | 11 | **Total** |
| --- | --- | --- | --- | --- | --- | --- | --- | --- | --- | --- | --- | --- |
| Bomback et al. (2010)[16] | Y^a^ | U^b^ | Y | Y | Y | Y | N^c^ | Y | U | U | Y | 7/11 |
| Dalbeth et al. (2015)[17] | Y | U | Y | Y | Y | Y | U | Y | U | U | N | 6/11 |
| Teng et al. (2013)[20] | Y | Y | Y | Y | Y | Y | U | Y | U | U | N | 7/11 |
| Bae et al. (2014)[21] | Y | U | Y | Y | Y | Y | U | Y | U | U | N | 7/11 |
| Meneses-Leon et al. (2014)[22] | Y | Y | Y | Y | Y | Y | Y | Y | N | U | N | 9/11 |
| Qian-Hua Li（2022[23] | Y | Y | Y | Y | Y | U | Y | Y | Y | U | N | 8/11 |
| W-T Lin et al. (2013）[24] | Y | Y | Y | Y | Y | U | Y | Y | Y | Y | N | 9/11 |
| Lee et al. (2024)[25] | Y | Y | Y | Y | Y | U | Y | Y | Y | U | N | 8/11 |
| Zheng et al. (2018)[28] | Y | Y | Y | Y | Y | U | Y | Y | Y | U | N | 8/11 |
| Siqueira et al. (2018）[29] | Y | Y | Y | Y | Y | U | Y | Y | Y | U | N | 8/11 |
| Joong Seob Lee[33] | Y | Y | Y | Y | Y | U | Y | Y | Y | U | N | 8/11 |
| S0n et al. (2010)[35] | Y | Y | Y | Y | Y | U | Y | Y | Y | U | N | 8/11 |
| Zhang et al. (2020)[37] | Y | Y | Y | Y | Y | Y | Y | Y | Y | U | N | 9/11 |
| Lin et al. (2021)[39] | Y | Y | Y | Y | Y | Y | Y | Y | Y | U | N | 9/11 |
| So, M. W(2020)[40] | Y | Y | Y | Y | Y | Y | U | Y | U | Y | N | 8/11 |

Note: Yes =Y^a^; No =N^c^; Unclear = U^b^; Agency for Healthcare Research and Quality (AHRQ*) checklist:(1) Define the source of information (survey, record review); (2) List inclusion and exclusion criteria for exposed and unexposed subjects (cases and controls) or refer to previous publications; (3) Indicate time period used for identifying patients; (4) Indicate whether subjects were consecutive if not population based; (5) Indicate if evaluators of subjective components of study were masked to other aspects of the status of the participants; (6) Describe any assessments undertaken for quality assurance purposes (e.g., test/retest of primary outcome measurements); (7) Explain any patient exclusions from analysis; (8) Describe how confounding was assessed and/or controlled; (9) If applicable, explain how missing data were handled in the analysis; (10) Summarize patient response rates and completeness of data collection; and (11) Clarify what follow‐up, if any, was expected and the percentage of patients for which incomplete data or follow‐up was obtained.

**Table 6.** The quality assessment of cohort study and case-control study.

| Study | Year | Selection | Comparability | Outcome | Total |
| --- | --- | --- | --- | --- | --- |
| Choi,H et al. | 2008 | *** | ** | *** | 8 |
| Choi,H et al. | 2010 | *** | ** | *** | 8 |
| Siqueira et al. | 2021 | *** | ** | *** | 8 |
| Zhang et al. | 2025 | ** | ** | *** | 7 |
| Meneses-León. et al. | 2020 | ** | ** | *** | 7 |
| Rai et al. | 2024 | *** | ** | *** | 8 |
| Bomback et al. | 2010 | *** | ** | *** | 8 |
| Batt et al. | 2014 | *** | ** | ** | 7 |

The NOS scale was used to evaluate the quality of the cohort study and case-control study.

**Supplementary Figure 1.** Sensitivity analysis of SSB and Hyperuricemia risk.

**Supplementary Figure 2.** Sensitivity analysis of Fructose and Hyperuricemia risk.

#

**Supplementary Figure 3.** Sensitivity analysis of FJ and gout risk.

A B

**C**

**Supplementary Figure 4.** Funnel plot of Hyperuricemia risk, (A) SSB, (B) FJ, (C) Fructose

A B

**C D**

**Supplementary Figure 5.** Funnel plot of G risk, (A) SSB, (B) FJ, (C) DSD, (D) Fructose
